# Editorial Note: Expression and function of the miR-143/145 cluster *in vitro* and *in vivo* in human breast cancer

**DOI:** 10.1371/journal.pone.0352941

**Published:** 2026-07-02

**Authors:** The PLOS One Editors

The *PLOS One* Editors issue this Editorial Note to inform readers that works cited in this article [1] as References 15 and 24 were retracted after the article’s publication date. PLOS considers that these references are not crucial in supporting the research or conclusions reported in [1].
